# Audit of Emergency Laparoscopic Cholecystectomy in a District General Hospital

**DOI:** 10.7759/cureus.50250

**Published:** 2023-12-09

**Authors:** Ayokunle Adenipekun, Amr Ibrahim Shalaby

**Affiliations:** 1 Surgery, Health Education England, Birmingham, GBR; 2 General Surgery, University Hospitals Birmingham NHS Foundation Trust, Birmingham, GBR

**Keywords:** upper gi tract, quality improvement projects, clincal audit, laporoscopic cholecystectomy, gall bladder diseases and gallstones

## Abstract

Introduction

Acute gallstone diseases are common surgical emergencies, accounting for approximately one-third of emergency surgical admissions. Laparoscopic cholecystectomy is the standard choice of treatment for gallstone diseases and is currently one of the most commonly performed surgical procedures in the United Kingdom. Majority of these procedures are carried out as elective cases. National Institute of Clinical Excellence (NICE) guidelines and other upper gastrointestinal surgery specialty bodies encourage early emergency surgery in acute symptomatic gallstone disease. We assessed emergency laparoscopic cholecystectomies performed at Birmingham Heartlands Hospital, United Kingdom and compared the practice against NICE and British Benign Upper Gastrointestinal Surgery Society (BBUGSS) recommendations.

Methods

This is a snapshot retrospective audit, assessing emergency laparoscopic cholecystectomy practice over a nine-month period from November 2022 to July 2023. Variables assessed were demographics, duration of symptoms prior to surgery, imaging modality, indications, C-reactive protein (CRP) levels, operative difficulty, intraoperative and postoperative complications, length of hospital stay and readmission rates. These variables were compared against both NICE and BBUGSS standards. We aimed to establish baseline data to encourage emergency laparoscopic cholecystectomies in our hospital and reduce repeated hospital visits for patients with acute gallbladder disease.

Results

Forty-eight patients had emergency laparoscopic cholecystectomy in the period reviewed, mean age was 44.3 years and females accounted for approximately 71% (n=34) of the group. 66.7% (n=32) of patients had their surgery within seven days of diagnosis with acute gallstone disease; 50% (n=24) of patients had no adverse intraoperative event. No patient had biliary tract injury despite a high number of difficult cases. Overall there was no correlation between duration before surgery and intraoperative difficulty or readmission rates.

## Introduction

Acute gallstone diseases are common surgical emergencies, accounting for approximately one-third of emergency surgical admissions [1]. Laparoscopic cholecystectomy (LC) is the standard surgical treatment of symptomatic gallstone disease. It is one of the most performed surgical procedures in the United Kingdom, with about 70,000 cases performed yearly [1]. Despite its popularity, these procedures can range in difficulty from routine to incredibly challenging even for very experienced surgeons.

The National Institute of Clinical Excellence (NICE) recommends that patients with acute cholecystitis should have laparoscopic cholecystectomy within seven days of diagnosis, and preferably within the same admission for those with gallstone pancreatitis [2]. The majority of Trusts across the United Kingdom perform LC via elective and ambulatory pathways, with only about 16% of LCs performed as an emergency [3,4]. This means patients are more likely to have repeated admissions and increased morbidity before elective surgery. Early laparoscopic cholecystectomies are associated with reduced complications, lower patient morbidity and reduced costs on the health system [3,5].

This audit assesses emergency LCs performed in a district general hospital in the West Midlands, England. The practice was compared against NICE recommendations on operative timing whilst complications and outcomes were compared against the British Benign Upper Gastrointestinal Surgery Society (BBUGSS) recommendations [6]. We aim to establish baseline data to encourage emergency LCs in our hospital and reduce repeated hospital visits for patients with acute gallbladder disease.

## Materials and methods

This is a snapshot retrospective audit of emergency laparoscopic cholecystectomies performed at Birmingham Heartlands Hospital, United Kingdom between November 2022 and July 2023. Patient demographics, clinical history and operative details were collected from electronic records. All patients who had laparoscopic cholecystectomy during an emergency admission within the period were included. These patients were admitted via the emergency department, surgical assessment unit or acutely referred from other in-hospital specialties. The procedures were carried out on a joint emergency theatre list and not separate hot gallbladder theatre lists. Exclusion criteria included non-emergency cases. Standard laparoscopic approach was used in all cases.

The audit was registered with the hospital's Clinical Audit Registration System and permission was granted to access patient data. All laparoscopic cholecystectomies included were carried out either by consultants or registrars with direct supervision from consultants. Pneumoperitoneum was attained with CO2 either via Modified Hassan's technique or Veress needle insufflation, depending on surgeon's preference. Following standard port insertions and divisions of adhesions when encountered, the gallbladder was identified. On attainment of critical view of safety, surgical or Hem-o-lok clips were applied to both the cystic artery and cystic duct. The gallbladder was dissected off the liver bed with strict haemostasis and retrieved via the umbilical incision. In cases with difficult dissections, oozing at the end of the procedure or concerns about a bile leak, a surgical drain was left in the sub-hepatic fossa. Subtotal cholecystectomies were performed in cases where it was deemed unsafe to dissect off the gallbladder and conversion to open surgery was completed via a Kocher incision. 

Variables assessed include age, gender, body mass index (BMI), investigations, duration of symptoms prior to surgery, indications, National Early Warning Score (NEWS), C-reactive protein (CRP) levels, operative difficulty, intraoperative and postoperative complications, length of hospital stay and readmission rates. The standards used were NICE guidelines which recommended surgery within seven days of diagnosis of acute cholecystitis [2] and BBUGSS outcome standards. These standards include >35% of patients having laparoscopic cholecystectomy within seven days, 30-day readmission rates <10%, conversion to open rates <5%, retained common bile duct (CBD) stones <2.5% and <1.5% for bile leak [5].

Data was entered into Microsoft Excel sheets and patient-identifying data was anonymised. Following data cleaning (removing duplicate entries, confirming inclusion criteria and appropriate coding), descriptive and statistical analyses of variables were carried out using Statistical Packaging for Social Sciences (SPSS) version 29.0 (IBM Corp., Armonk, NY, USA). Statistical analysis was completed using Chi-square test for categorical variables and Mann-Whitney U Tests for assessing differences between groups. The p-value for significance was set at 0.005. 

## Results

**Figure 1:**
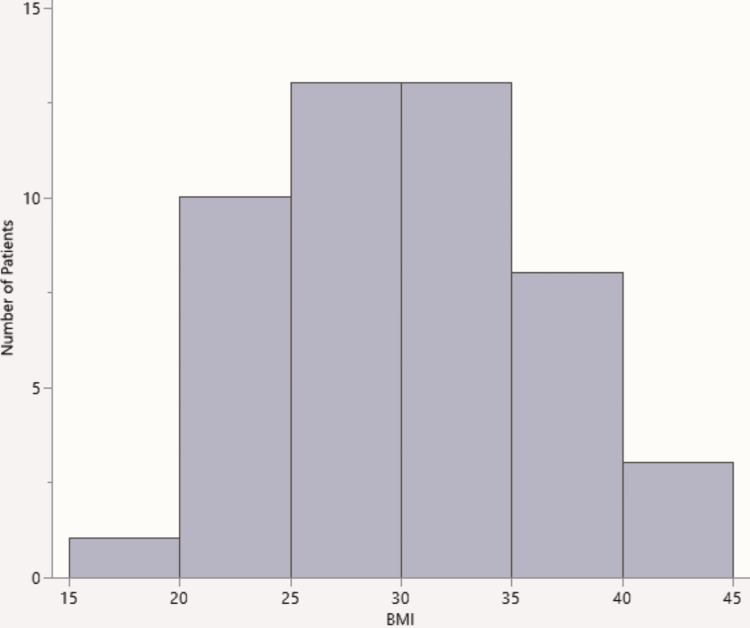
Distribution of patients' BMI BMI: Body Mass Index.

**Figure 2:**
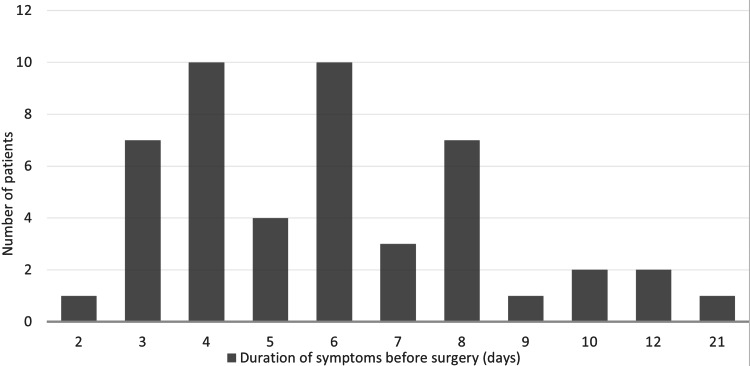
Description of duration of symptoms before emergency surgery.

Forty-eight emergency LCs were performed in the nine-month period. The average age was 44.3 years (SD=18.4). The youngest patient was 17, whilst the oldest was 85 years. The majority of the patients were fit, approximately 45.8% (n=22) were American Society of Anaesthesiologists (ASA) grade 1, only four patients (8.33%) were ASA 3, and no one had an ASA grade more than 3 (Table 1). The median BMI was 29.45 (interquartile range (IQR) 8.91) (Figure 1). The average duration prior to LC was 6.1 days (SD=3.3). Approximately 66.7% (n=32) of patients had LC within seven days (Figure 2). 

**Table 1:** Summary of patient details ASA: American Society of Anaesthesiologists.

Patient demographics	Number of patients
Age (years)	Patients (N)
17-26	10
27-36	9
37-46	10
47-56	7
57-66	5
67-76	3
77-86	4
Total	48
Gender	Patients (N)
Male	14
Female	34
Total	48
ASA	Patients (N)
Grade 1	22
Grade 2	22
Grade 3	4
Total	48

Seventy-nine percent (n=38) of all patients had at least an ultrasound scan in the index admission. Other imaging modalities used were Computerized Tomography (CT) scans and Magnetic Resonance Cholangio-Pancreatography (MRCP). Throughout their in-patient preoperative period, only seven patients had a NEWS greater than 5. CRP values ranged from 1 to 631, mean CRP was 130.8 (SD=156.41). The most common indication for surgery was acute cholecystitis, closely followed by severe biliary colic. Only 4.2% (n=2) of patients had pancreatitis alone. Intraoperative difficulty was accessed using Nassar difficulty grading system [7]; grades 1 and 4 occurred most frequently (Table 2).

**Table 2:** Description of clinical and perioperative variables. USS: ultrasound scan, CT: computerized tomography, MRCP: magnetic resonance cholangiopancreatography, CBD: common bile duct, NEWS: National Early Warning Scores

Clinical and perioperative details	Number of patients
Imaging	Patients(N)
USS alone	15
CT alone	4
MRCP alone	2
USS and CT	3
USS and MRCP	13
CT and MRCP	3
CT + MRCP + USS	7
None	1
Total	48
Indications	Patients (N)
Acute cholecystitis	32
Biliary colic	9
Pancreatitis	2
CBD stone + biliary colic	1
Pancreatitis + biliary colic	2
Pancreatitis + cholecystitis	2
Total	48
Nassar Difficulty	Patients(N)
Grade I	14
Grade II	9
Grade III	11
Grade IV	14
Total	48
NEWS	Patients(N)
Score <5	41
Score ≥5	7
Total	48

Fifty percent (n=24) of patients had no adverse intraoperative event. Approximately 21% (n=10) had bile spillage, stone spillage and drain insertion. There was no common bile duct or bowel injury (Table 3).

**Table 3:** Description of intraoperative events

Intraoperative events	Patients (N)
Bile spillage only	3
Split stones only	1
Drain insertion only	6
Bile spillage + split stone	1
Bile spillage + drain insertion	3
Bile spillage + split stone + drain insertion	10
No adverse outcome	24
Total	48

**Figure 3:**
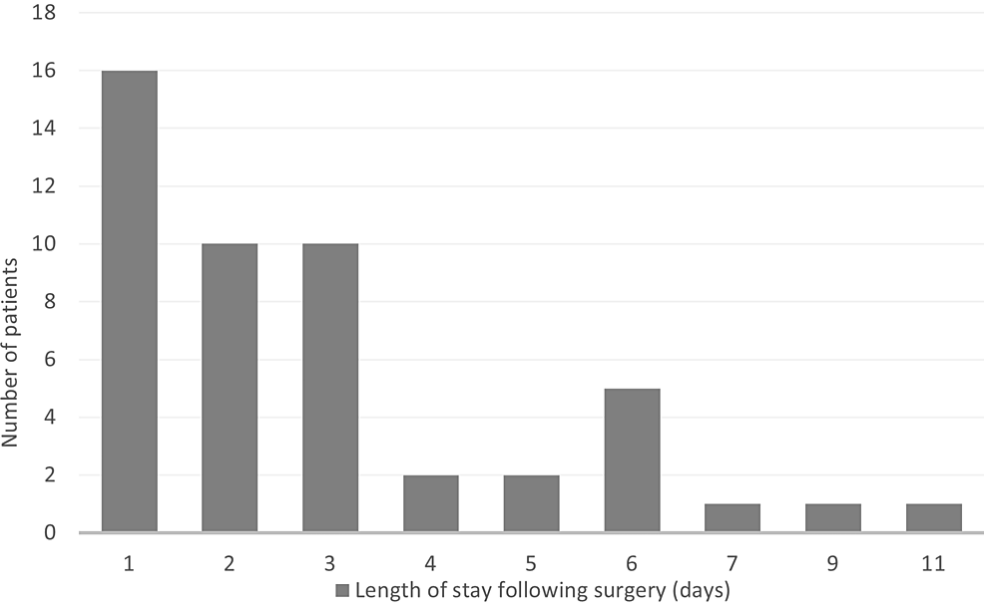
Length of stay following completion of emergency laparoscopic cholecystectomy

Our emergency LC completion rate was 87.5% (n=42). Six (12.5%) patients had subtotal laparoscopic cholecystectomy and of this group, there was one conversion to open surgery. One patient returned to theatre due to a mesenteric bleed and one patient had retained common bile duct stone. A third of the patients had an overnight stay following LC and by the end of the second postoperative day, 54.2% (n=26) of patients had been discharged. The average length of stay following surgery was 2.9 days (SD=2.3) (Figure 3). There were three readmissions within 30 days, for diarrhoea, retained CBD stone and bile leak from the liver bed.

Chi-square test didn’t reveal any correlation between the duration of symptoms (<7 or ≥7 days) and intraoperative difficulty, X2 (3, N = 48) = 1.7, p=0.698. The readmission rates also did not differ by the duration of symptoms, X2 (1, N = 48) = 1.712, p=0.191.

Mann-Whitney U test was used to assess if length of stay following surgery differed by duration of symptoms prior to emergency surgery. There was a shorter length of stay following surgery for patients who had their procedure after seven days. This difference was not statistically significant (z=-0.855; p=0.392).

## Discussion

Patient demographics were like other established epidemiological data on gallstones related disease, with higher prevalence in females and association with increased BMI [8,9]. Approximately 71% (n=34) of these patients had cholecystitis; this is a considerably higher percentage than the commonly quoted prevalence for cholecystitis, which is about 10% of symptomatic gallstone disease [1]. This may be explained by the fact that many surgical firms are more likely to perform emergency LCs on patients with cholecystitis than only on biliary colic, although the Association of Upper Gastrointestinal Surgery of Great Britain and Ireland (AUGIS) recommends surgeons perform LC in persisting biliary colic [10]. About 18.75% (n=9) of patients who underwent emergency LC had only biliary colic.

NICE guidelines recommend LCs be done within a week for acute cholecystitis [2], AUGIS further recommends LCs to be done within 72 hours of index admission for acute cholecystitis [10]. Although the majority of patients in our study had their LCs within seven days, the mean duration was closer to seven than three. This may be attributed to factors such as delayed presentations, multiple investigations and limited theatre resources. Overall, our performance exceeded the standards set by BBUGSS which recommends that at least 35% of patients with acute cholecystitis have LC within seven days [6]. 

Despite the concerns about the hostility of an inflamed biliary system, early cholecystectomies have been shown to improve outcomes [11,12]. A large United Kingdom-based cohort study showed reduced complications and length of stay when LCs were done within three days [5]. The Society of American Gastrointestinal and Endoscopic Surgeons (SAGES) reassures surgeons that early LCs done within 24-72 hours are not associated with adverse outcomes [13]. Most of the patients included in the audit had Grade 4 Nassar intraoperative difficulty, however there were no bowel or bile duct injuries. There was a 2.1% (n=1) conversion to open rate and our 30-day readmission rate was 6.3% (n=3). Similarly, a study by Knight et al. demonstrated that timings of laparoscopic cholecystectomies did not have an impact on conversion rate [14]. There was one readmission with a retained CBD stone, accounting for 2.1% (n=1) of patients. Comparing these outcomes with BBUGSS standard [6], we identified good performance with readmission rates <10%, conversion to open rates <5% and <2.5% for retained CBD stones within 90 days. However, only one patient (2.1%) had bile leak from an aberrant duct near the liver bed; this was higher than the BBUGSS recommendation of <1.5% bile leak rate after surgery.

Early LCs are related to shorter postoperative length of stay [5,12,15], however our audit showed a shorter length of stay in patients who had emergency LCs after seven days. Although this difference was not statistically significant, sub-analysis of this cohort showed all 16 patients in this group had ASA 1 or 2 only suggesting their preoperative state may be contributory. This will be further explored in a larger re-audit.

Limitations of this audit include a relatively small sample size over the period reviewed, varying skill levels between surgeons and patient recall bias in determining onset of symptoms. We aim to address these limitations with a further re-audit over a longer period, with a larger sample size and decreasing interoperative variability by analysing surgical outcomes in specific homogenous groups like using number of previous laparoscopic cholecystectomies performed or job grades (consultant, higher specialty trainees and core trainees). 

## Conclusions

This audit demonstrates safe completion of emergency laparoscopic cholecystectomy compared against chosen standards despite high level of intraoperative difficulty. However, the scope to improve postoperative length of stays and quicker time to emergency surgery exists. The establishment of increased local awareness and developing pathways to encourage same-admission emergency laparoscopic cholecystectomy pathways will improve this practice and positively impact patient journey, therefore decreasing morbidity associated with gallstone disease.
